# Code-free automated machine learning for OCT-based classification of vitreoretinal interface diseases

**DOI:** 10.1186/s40942-026-00880-9

**Published:** 2026-06-08

**Authors:** Lorenzo Ferro Desideri, Enrico Bernardi, Carla Troyas, Yousif Subhi, Mehmet Cem Sabaner, Martin Zinkernagel, Rodrigo Anguita

**Affiliations:** 1 Department of Ophthalmology, Inselspital, Bern University Hospital, University of Bern, Bern, Switzerland; 2 Bern Photographic Reading Center, Inselspital, Bern University Hospital, University of Bern, Bern, Switzerland; 3 Moorfields Eye Hospital NHS Foundation Trust, London, UK; 4 School of Medicine and Health, Technical University of Munich, Munich, Germany; 5 Department of Ophthalmology, Rigshospitalet, Glostrup, Denmark; 6 Department of Ophthalmology, University Hospital of Southern Denmark, Sønderborg, Denmark; 7 Department of Regional Health Research, University of Southern Denmark, Odense, Denmark; 8 Department of Ophthalmology, Kastamonu University, Training and Research Hospital, Kastamonu, 37150 Turkey

**Keywords:** Optical coherence tomography, Automated machine learning, Vitreoretinal interface, Macular hole, Epiretinal membrane, Image classification

## Abstract

**Background:**

Differentiation of vitreoretinal interface disorders on optical coherence tomography (OCT) relies on expert interpretation and can be challenging in borderline cases. Automated machine learning (AutoML) platforms may enable clinician-driven artificial intelligence development without coding expertise. This study evaluated the performance of a code-free AutoML approach for OCT-based classification.

**Methods:**

In this cross-sectional image classification study, 434 OCT B-scans from publicly available datasets were manually labeled into four categories: epiretinal membrane (ERM), lamellar macular hole (LMH), full-thickness macular hole (MH), and normal retina. Images were uploaded to a cloud-based AutoML platform (Google Cloud Vertex AI), which automatically performed data splitting (80% training, 10% validation, 10% test), model training, and optimization. Performance was assessed using precision, recall, average precision, and confusion matrix analysis.

**Results:**

The model achieved an overall average precision of 0.988, with precision and recall of 97.6%. MH and normal retina were classified with perfect precision and recall (100%). ERM showed high precision (100%) with slightly reduced recall (92.9%), while LMH demonstrated complete recall (100%) with lower precision (83.3%). Misclassifications were limited to anatomically related entities.

**Conclusions:**

Code-free AutoML enables accurate OCT-based classification of vitreoretinal interface disorders using a clinician-driven workflow. This approach may facilitate broader adoption of artificial intelligence in ophthalmology and support rapid clinical research prototyping.

## Background

In routine clinical practice, differentiation between vitreoretinal interface disorders such as full-thickness macular hole (MH), lamellar macular hole (LMH), and epiretinal membrane (ERM) is primarily based on optical coherence tomography (OCT) morphology [1–3]. While advanced cases are usually straightforward, borderline presentations and overlapping structural features are frequently encountered, particularly in early disease stages or mixed phenotypes [4]. In these settings, diagnostic classification relies heavily on expert interpretation and subtle morphological details rather than on predefined criteria.

In parallel with advances in retinal imaging, artificial intelligence (AI) has gained increasing attention in ophthalmology and vitreoretinal diseases, especially for image-based classification tasks [5–8]. Conventional machine learning and deep learning approaches have shown promising results but often require solid expertise in data preprocessing, feature engineering, and model optimization [7]. This technical complexity may limit accessibility and adoption among ophthalmologists without formal training in data science [9]. 

In this perspective, automated machine learning (AutoML) platforms have been developed to address these limitations with the code-free automatization of the main steps leading to model development, including data splitting, feature extraction, architecture selection, and hyperparameter tuning [10, 11]. AutoML allows clinicians to explore AI-based approaches focusing on clinically relevant questions rather than algorithmic design.

In this study, we evaluated whether a clinician-developed, code-free AutoML workflow could achieve accurate single-label classification of common vitreoretinal interface disorders on OCT. The aim was not to propose a replacement for expert clinical interpretation, but to assess the feasibility and performance of a pragmatic AutoML approach consistent with real-world clinical research workflows.

## Methods

### Dataset and image labeling

The images were taken from two publicly available, open-access datasets of retinal OCT B-scan images: the Kaggle Retinal OCT C8 dataset and the OCTDL dataset [12].The dataset included OCT images representing common vitreoretinal interface disorders as well as normal retinal anatomy. All images were fully anonymized and used exclusively for research purposes. This study used publicly available, fully anonymized datasets and did not involve identifiable human participants. Therefore, ethics approval and informed consent were not required according to institutional guidelines.

All OCT scans were manually labeled by 2 expert retinal specialists (LFD and RA) according to the underlying diagnosis and assigned to one of four classes: epiretinal membrane (ERM), lamellar macular hole (LMH), full-thickness macular hole (MH), or normal retina. Disagreements were resolved by consensus.

**Fig. 1:**
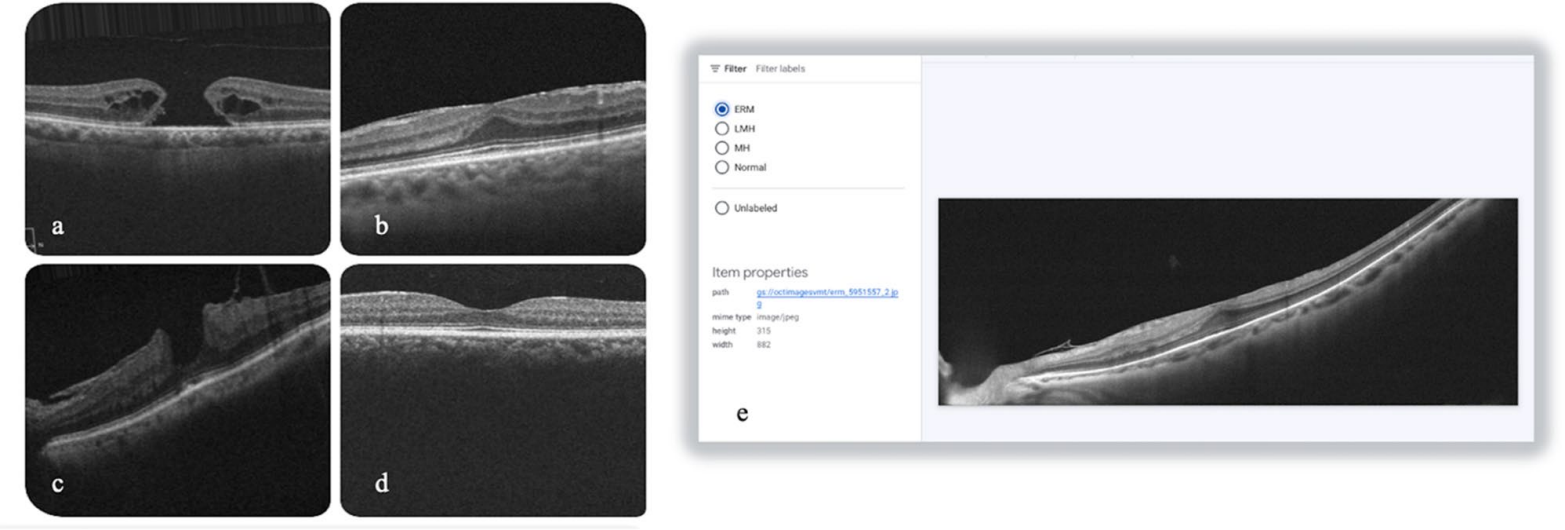
Representative OCT classes and single-label annotation workflow. Representative OCT B-scans illustrating the four classification categories used for model training: (**a**) full-thickness macular hole (MH), (**b**) lamellar macular hole (LMH), (**c**) epiretinal membrane (ERM), and (**d**) normal retina. Panel (**e**) shows an example of manual single-label annotation on the AutoML platform, where each OCT image is assigned to a single diagnostic category (ERM, LMH, MH, or normal) based on clinical judgment, forming the supervised training dataset

Each image received a single diagnostic label, reflecting routine clinical OCT interpretation and enabling a fully supervised learning approach. Single fovea-centered OCT B-scans were used for model training and evaluation, with each B-scan treated as an independent sample, as patient-level linkage was not available. Representative OCT examples and the single-label annotation workflow are shown in Fig. 1.

**Fig. 2:**
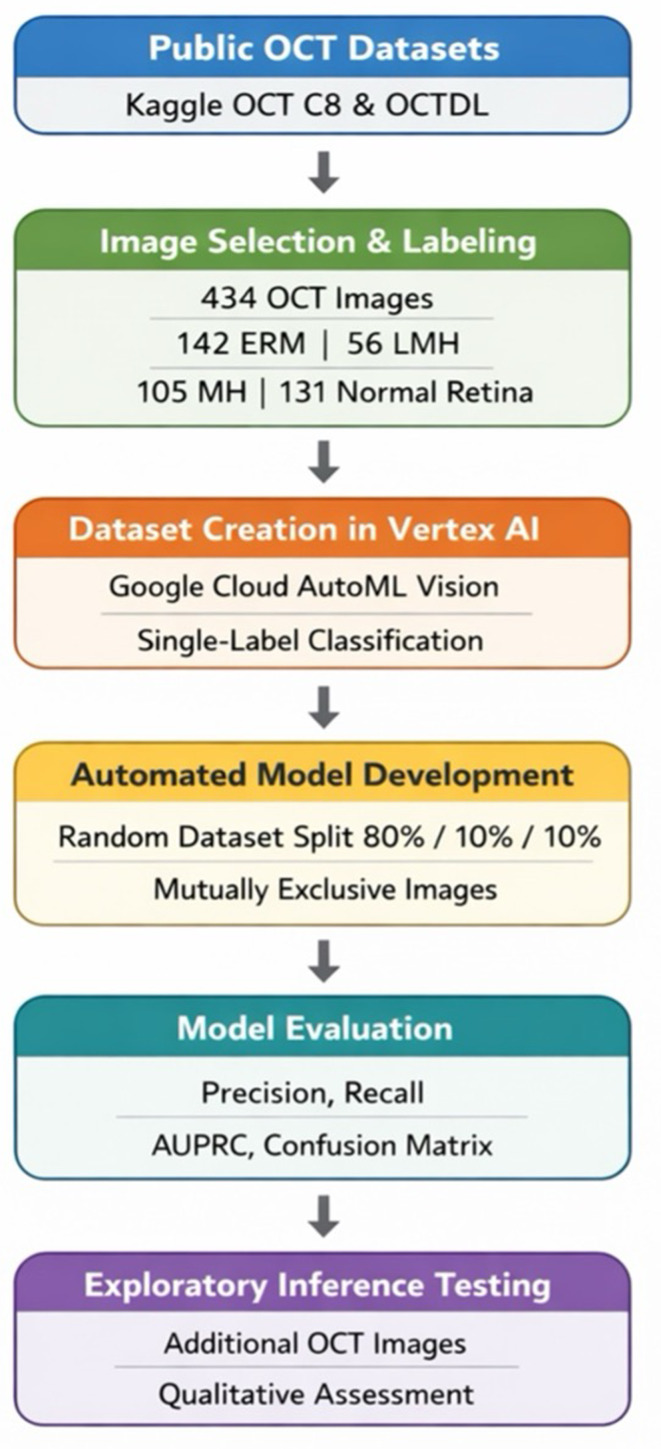
AutoML workflow for OCT-based classification of vitreoretinal interface disorders. Flowchart illustrating the study pipeline. OCT B-scans from two public datasets (Kaggle OCT C8 and OCTDL) were manually labeled into four categories (ERM, LMH, MH, normal retina) and uploaded to the Google Cloud Vertex AI AutoML platform. The system automatically performed dataset splitting (80% training, 10% validation, 10% test) and model development. Model performance was evaluated using precision–recall metrics and confusion matrix analysis

**Fig. 3:**
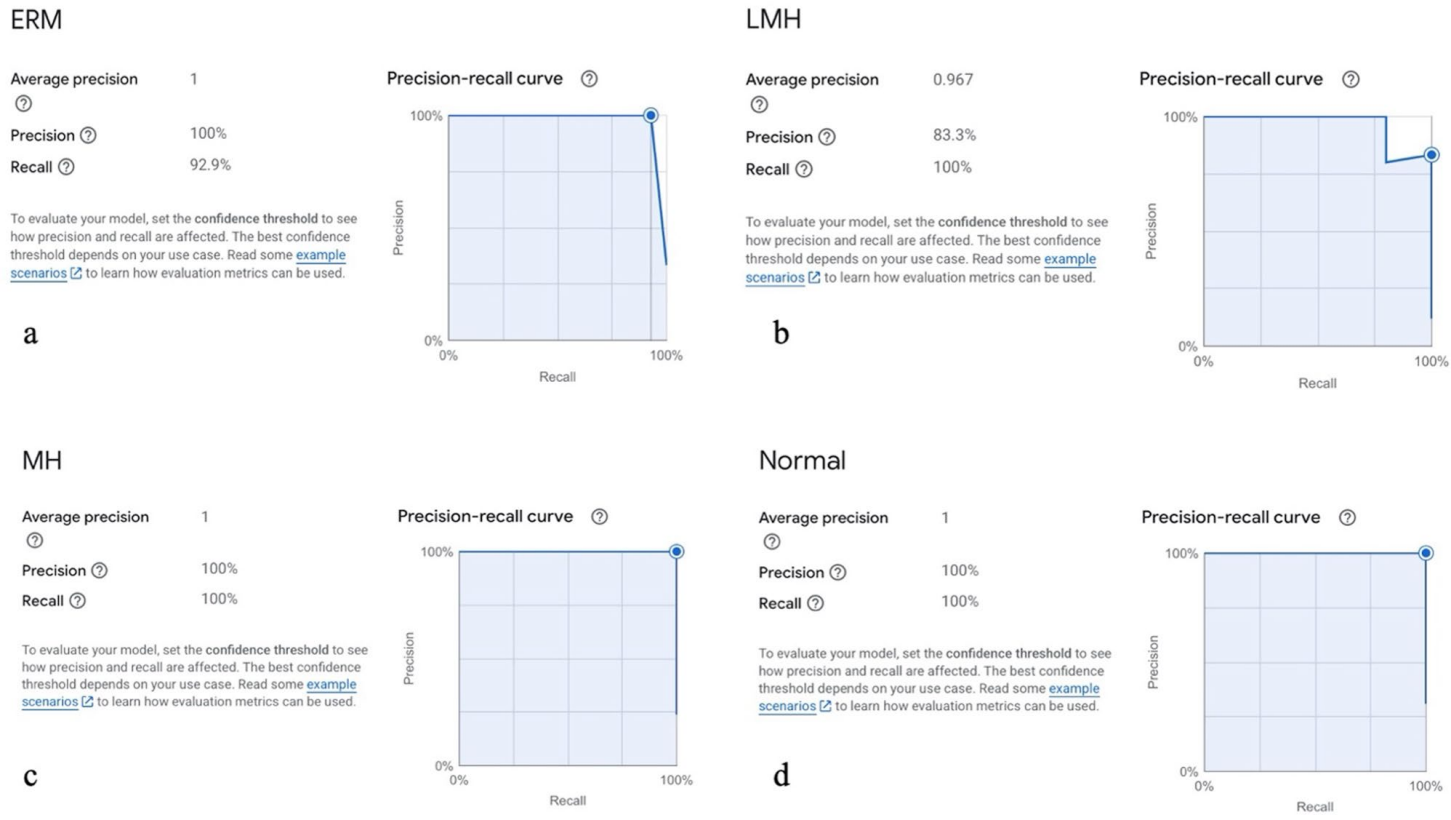
Class-specific performance of the AutoML model. Precision–recall curves and summary metrics for each diagnostic category: (**a**) epiretinal membrane (ERM), (**b**) lamellar macular hole (LMH), (**c**) full-thickness macular hole (MH), and (**d**) normal retina. MH and normal retina achieved perfect precision and recall, while ERM and LMH showed slightly reduced performance, reflecting greater morphological overlap at the vitreoretinal interface

**Fig. 4:**
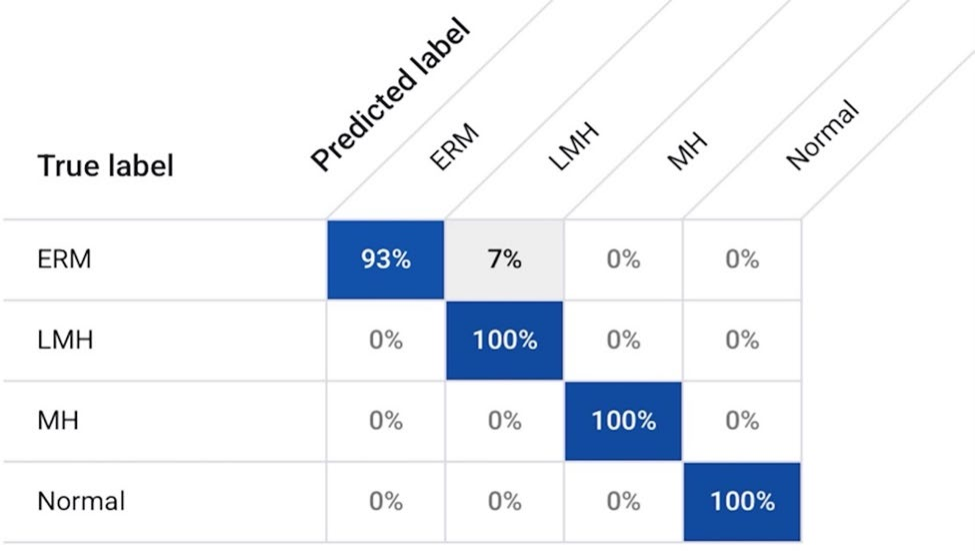
Confusion matrix of the AutoML model for vitreoretinal interface disorders. The model achieved complete recall for LMH, MH, and normal OCT images, while minor misclassification was observed for ERM, primarily toward LMH, reflecting morphological overlap between these entities

**Fig. 5:**
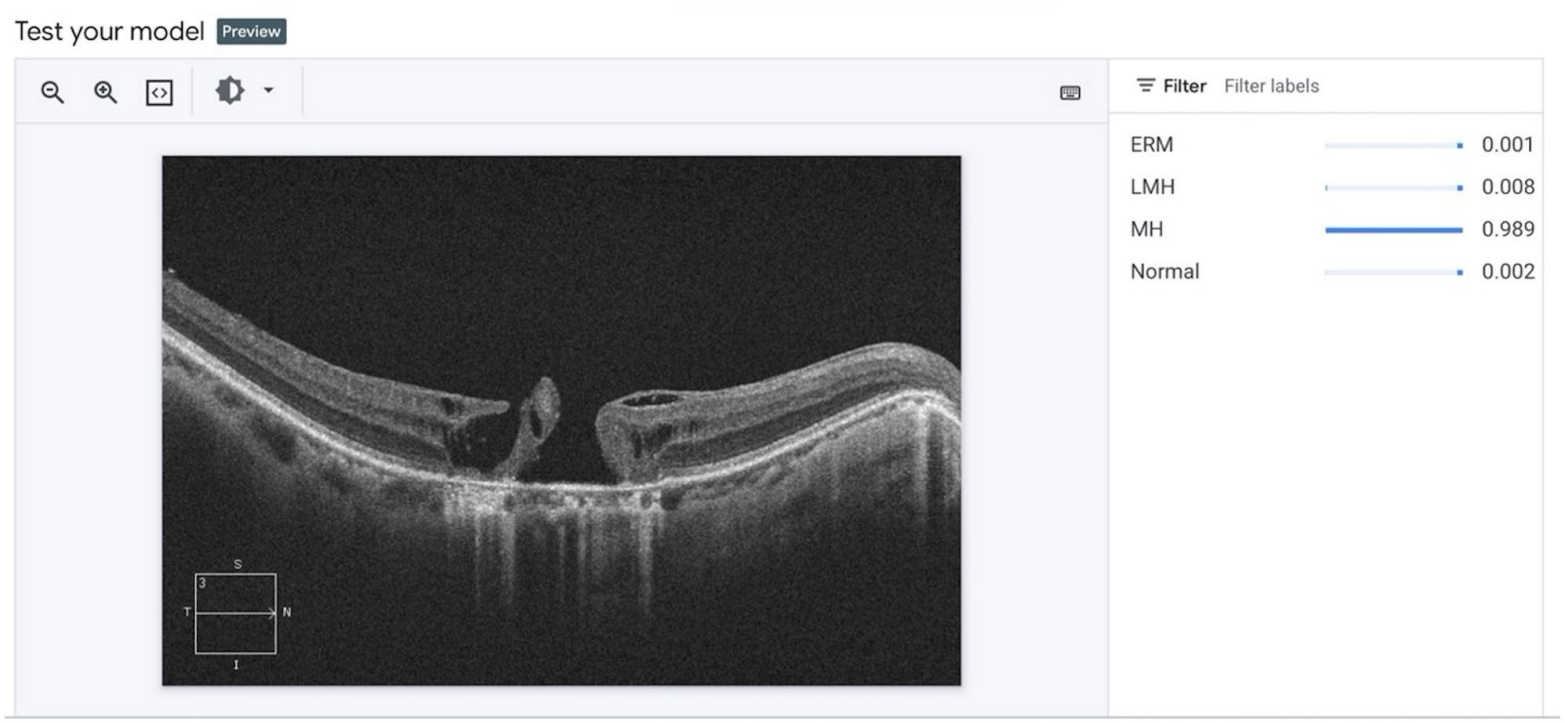
Exploratory qualitative inference example on a held-out OCT image. The AutoML model correctly classified the OCT scan as macular hole (MH) with high confidence (0.989). Probability scores for the remaining classes (ERM, LMH, and normal) were low, illustrating class separation at inference time

### AutoML model development

OCT images were uploaded to a cloud-based automated machine learning platform (Google Cloud Vertex AI) to create a single-label image classification dataset. No manual image preprocessing, normalization, feature extraction, or data augmentation was applied. The workflow was intentionally designed to reflect a pragmatic, clinician-driven research setting with minimal technical intervention.

The AutoML Vision framework automatically evaluates multiple convolutional neural network architectures and selects the optimal model configuration during training. Incremental training was not enabled, and the model was trained from scratch. Model architecture selection, feature extraction, and hyperparameter optimization were fully managed by the platform without user-defined customization.

Model training was performed using the platform’s automated image classification pipeline. Model development, including architecture selection, feature extraction, hyperparameter optimization, and training configuration, was fully managed by the platform without user-defined customization. The exact underlying architecture and training parameters were not directly accessible, reflecting the closed-box nature of the commercial AutoML workflow.

The dataset was automatically partitioned into training (80%), validation (10%), and test (10%) subsets using the platform’s default random sampling settings.

To minimize the risk of data leakage, images included in the training, validation, and test sets were mutually exclusive, and no image used for model evaluation was seen by the model during training.

### Model evaluation and statistical analysis

Model performance was evaluated on the held-out test set at a confidence threshold of 0.5 using the native evaluation pipeline provided by the Google Cloud Vertex AI AutoML platform. Evaluation metrics included overall and class-specific precision (positive predictive value), recall (sensitivity), average precision, and confusion matrix analysis. Performance estimates were automatically generated by the platform without additional post hoc statistical modeling.

Additional qualitative testing was performed using OCT images from the same publicly available datasets that were not included in the training, validation, or test sets. These images were used only for exploratory qualitative assessment of model behavior and were not included in quantitative performance analysis (Fig. 2).

## Results

A total of 434 OCT images were included in the final dataset, comprising 142 ERM, 56 LMH, 105 full-thickness MH and 131 normal retina images.

Precision reached 100% for MH and normal retina. ERM showed 100% precision with slightly reduced recall (92.9%), while LMH exhibited high overall performance despite lower precision (83.3%), likely reflecting morphological overlap with ERM. **(**Table 1; Fig. 3). Errors were infrequent and largely confined to anatomically related entities at the vitreoretinal interface, suggesting that misclassification reflected intrinsic morphological ambiguity rather than systematic model bias. The corresponding confusion matrix is presented in Fig. 4. Importantly, no systematic confusion was observed between normal and pathological OCT images, indicating reliable separation between healthy retinal anatomy and disease.

**Table 1:** Performance of the AutoML model for vitreoretinal interface disorder classification

Retinal Condition	Average Precision (AUPRC)	Sensitivity (Recall) (%)	PPV (Precision) (%)
Aggregate	0.988	97.6	97.6
Full-thickness macular hole (MH)	1.000	100	100
Lamellar macular hole (LMH)	0.967	100	83.3
Epiretinal membrane (ERM)	1.000	92.9	100
Normal OCT	1.000	100	100

Class-specific and aggregate average precision (AUPRC), sensitivity (recall), and positive predictive value (precision) for full-thickness macular hole (MH), lamellar macular hole (LMH), epiretinal membrane (ERM), and normal OCT images

Model performance remained stable across a range of confidence thresholds, with high precision preserved at the selected threshold of 0.5, corresponding to an overall precision of 97.6% and recall of 97.6%. When evaluated on additional OCT images from the same publicly available datasets that were not included in the training, validation, or test sets, the model maintained good qualitative classification performance in most exploratory cases (Fig. 5).

## Discussion

In this study, we demonstrated that a simple clinician-developed, code-free AutoML workflow can show accurate and reliable classification of vitreoretinal interface disorders by OCT imaging. Our model achieved overall satisfying precision and recall through the adoption of a fully automated pipeline based on supervised clinical labeling, with particularly strong performance for full-thickness MH and normal OCT images.

The excellent performance observed for MH and normal retina is clinically intuitive and consistent with prior deep learning literature [13, 14]. These categories are characterized by distinct and visually salient OCT features, including a full-thickness foveal defect in MH and the absence of pathological alterations in normal eyes, which can be readily captured by automated feature extraction [15]. In fact, previous OCT-based deep learning studies have similarly reported higher performance for conditions with clearly defined structural hallmarks compared with entities exhibiting more subtle morphological changes [16–18]. Along similar lines, Durmaz Engin et *al.* compared AutoML with an expert-designed deep learning model for vitreomacular interface disorders and found excellent performance for normal retina and full-thickness MH, while ERM and LMH showed lower recall in the AutoML model, consistent with the intrinsic morphological overlap of these conditions [10]. 

In this regard, classification performance, although still very satisfying, was slightly lower for ERM and LMH, particularly in terms of recall values. As postulated above, these findings may likely reflect an intrinsic higher diagnostic complexity of these disorders, due to the presence of overlapping OCT features especially in early phases of the disease. In fact, the clinical differentiation between ERM and early LMH, or from tractional changes without full-thickness disruption, can be challenging even among experienced clinicians [19, 20].

In a prior AutoML study on OCT-based retinal disease classification, ERM detection achieved an AUPRC of 0.978, with a precision of 92.9% and recall of 86.7%, values that are broadly comparable to the performance observed in our ERM cohort, supporting the reproducibility of AutoML approaches for this diagnostically challenging entity [11]. Nonetheless, the limited misclassifications observed in our study were largely confined to these anatomically related entities, likely reflecting simple morphological overlap rather than systematic model failure. Importantly, no misclassification occurred between pathological and normal OCT images, supporting robust separation between healthy and diseased retinal anatomy. Future studies should therefore consider multilabel classification strategies rather than single-label approaches, as vitreoretinal interface disorders represent a morphological spectrum in which multiple biomarkers frequently coexist within the same scan.

Furthermore, previous studies have applied AutoML to retinal disease classification using non-OCT imaging, including handheld fundus photography, ultra-widefield images, and B-scan ultrasound, with consistently strong performance across multiple pathologies [21–23]; however, these applications have mostly focused on surface-level retinal imaging, which differs substantially from OCT in terms of depth and structural detail. OCT remains the primary imaging tool for macular disease in everyday vitreoretinal practice [19], yet most OCT-based artificial intelligence studies still rely on custom deep learning models that require considerable technical expertise [24–26]. By contrast, evidence supporting AutoML for OCT imaging is still limited, despite its potential to simplify model development and support clinician-led research [10, 11].

Moreover, the aim of this study was not to outperform expert clinicians, but to evaluate the feasibility of a pragmatic, no-code AutoML approach that does not require formal training in programming or data science. In this context, the observed performance is notable. Unlike traditional deep learning workflows, the AutoML pipeline required no coding or manual feature engineering, with clinicians contributing primarily through image labeling and dataset curation tasks that closely reflect routine clinical practice. While training ophthalmologists in artificial intelligence remains important [9], this approach simplifies access to the field and facilitates understanding and adoption in everyday clinical research.

Importantly, the present findings demonstrate technical feasibility rather than real-world clinical utility. Prospective validation studies, integration into clinical imaging workflows, and comparison with current diagnostic pathways will be necessary before potential routine clinical implementation can be considered.

Several limitations should be acknowledged, including the modest dataset size and the use of publicly available OCT images, which may limit generalizability. An additional limitation of this study is the relatively small dataset and test-set size, particularly for lamellar macular hole. Individual misclassifications may therefore have had a disproportionate impact on precision and recall values. Accordingly, these findings should be interpreted primarily as exploratory proof-of-concept results rather than definitive measures of clinical performance.

Another important limitation is the potential risk of patient-level data leakage. Since patient identifiers were not available in the public datasets used, it was not possible to determine whether multiple B-scans from the same eye were distributed across training and test subsets. This may have artificially inflated performance estimates and should be considered when interpreting the results. Larger and more diverse datasets from multiple devices and centers will be needed for robust external validation. In addition, the single-label framework does not reflect the coexistence of multiple pathologies often seen in clinical practice, and the closed nature of commercial AutoML platforms limits model interpretability and customization.

Despite these limitations, our results support the feasibility of clinician-developed, code-free AutoML for OCT-based classification of vitreoretinal interface disorders. The strong performance achieved with minimal technical overhead highlights AutoML as a practical tool for clinician-led research and early clinical prototyping, with potential to complement specialist expertise following further validation.

## Conclusions

In conclusion, this study demonstrated that a simple, clinician-developed, code-free AutoML workflow can achieve accurate OCT-based classification of vitreoretinal interface disorders. By relying on supervised clinical labeling and fully automated model training, this approach offers a pragmatic entry point for clinician-led artificial intelligence research without the need for advanced technical expertise. While performance was highest for entities with distinct morphological features, results remained clinically meaningful across all classes. With further validation on larger, multicenter datasets, AutoML may represent a scalable tool to support research, education, and early clinical prototyping in vitreoretinal practice.

## Data Availability

The datasets used in this study are publicly available from the Kaggle Retinal OCT dataset and the OCTDL dataset. Additional data are available from the corresponding author upon reasonable request.
